# Multisource Coherence Analysis of the First European Multicenter Cohort Study for Cancer Prevention in People Experiencing Homelessness: Data Quality Study

**DOI:** 10.2196/73596

**Published:** 2025-11-14

**Authors:** Antonio Blasco-Calafat, Vicent Blanes-Selva, Tobias Fragner, Ascensión Doñate-Martínez, Tamara Alhambra-Borrás, Julia Gawronska, Lee Smith, Juan M Garcia-Gomez, Igor Grabovac, Carlos Sáez

**Affiliations:** 1 Biomedical Data Science Laboratory Institute of Information and Communication Technologies Universitat Politècnica de València Valencia Spain; 2 Department of Social and Preventive Medicine Center for Public Health Medical University of Vienna Vienna Austria; 3 Instituto de Investigación en Políticas de Bienestar Social Department of Social and Preventive Medicine Universitat de València Valencia Spain; 4 Center for Health, Performance and Wellbeing Anglia Ruskin University Cambridge United Kingdom

**Keywords:** homelessness, data quality, data variability, public health, data analysis, curated dataset, multisource

## Abstract

**Background:**

Coherence across sites in multicenter datasets is one substantial data quality dimension for reliable health data reuse, as unexpected heterogeneity in data can lead to biases in data analyses and suboptimal generalization of results.

**Objective:**

This work aims to characterize and label the data coherence across sites in the first European multicenter dataset for cancer prevention in people and early detection among the homeless population in Europe: coadapting and implementing the health navigator model. This dataset emerged to enable research to address disparities in health challenges and health care access due to barriers such as unstable housing, limited resources, and social stigma in people experiencing homelessness.

**Methods:**

The dataset comprises 652 cases: 142 from Austria, 158 from Greece, 197 from Spain, and 155 from the United Kingdom. All participants fit classifications from the European Typology of Homelessness and Housing Exclusion. This longitudinal study collected questionnaires at baseline, after 4 weeks, and at the end of the intervention. The 180-question survey covered sociodemographic data, overall health, mental health, empowerment, and interpersonal communication. Data variability was assessed using information theory and geometric methods to analyze discrepancies in distributions and completeness across the dataset.

**Results:**

Substantial variability was observed among the 4 pilot countries, both in the overall analysis and within specific domains. In particular, measures of health care empowerment, quality of life, and interpersonal communication demonstrated the greatest discrepancies among pilot sites, with the exception of the health domain. Notably, Spain exhibited the most pronounced differences, characterized by a high number of missing values related to interpersonal communication and the use of health care services.

**Conclusions:**

Health data may be comparable across the 4 countries; however, substantial differences were observed in the other questionnaires, requiring independent, country-specific analyses. This study underscores the heterogeneity among people experiencing homelessness and the critical need for data quality assessments to inform future research and policymaking in this field.

## Introduction

Homelessness is a complex and multifaceted phenomenon, encompassing more than the mere absence of a physical dwelling. It signifies a state of profound vulnerability and social exclusion, characterized by a lack of stable, safe, or adequate housing [1-3]. The experience of homelessness is deeply intertwined with a range of social, economic, and individual factors, including poverty, unemployment, mental health challenges, substance use, and domestic violence. People experiencing homelessness constitute a highly heterogeneous group with varying backgrounds, needs, and challenges, encompassing socioeconomic, cultural, physical, and mental health aspects, as well as historical and geographical contexts [4,5]. This inherent diversity within the group of people experiencing homelessness, coupled with inconsistent definitions and methodological variations in data collection across countries, makes it exceedingly challenging to accurately estimate the true prevalence of homelessness. Despite these complexities, it is estimated that at least 895,000 individuals in Europe are experiencing homelessness on any given night, living rough or in temporary or emergency accommodations [6].

Research consistently demonstrates substantial health disparities between people experiencing homelessness and the housed population. Homelessness is associated with poorer health outcomes, including premature mortality, partly due to barriers to accessing health care [7-9]. Furthermore, people experiencing homelessness experience higher cancer morbidity, lower cancer screening rates, later-stage cancer diagnoses, and poorer cancer-specific health outcomes compared to the housed population [10], with cancer being the second most common cause of death among people experiencing homelessness [11]. Among the most relevant factors, people experiencing homelessness usually report low health literacy, especially regarding cancer [12], and they tend to experience barriers to accessing fragmented cancer prevention and community health services [13].

Research interest in improving the health status and cancer prevention efforts for people experiencing homelessness is growing, leading to the implementation and piloting of innovative health care strategies, such as the patient navigation model and the patient empowerment model [14,15]. In the patient navigation model, patient navigators, defined as health care professionals or peers specializing in care coordination, case management, and reducing barriers to care, play a crucial role in guiding individuals to overcome obstacles and access health care services in a timely manner [16]. This includes facilitating access to preventive care and health promotion programs, which have been shown to improve their users’ health outcomes and care satisfaction [17]. The patient empowerment model focuses on equipping individuals with the knowledge, skills, and confidence to take an active role in managing their health and navigating the health care system [18,19]. This approach emphasizes self-efficacy and shared decision-making, empowering patients to make informed choices about their health care and advocate for their needs.

Within the CANCERLESS project, a multicenter study funded by the European Union, critical components of the patient navigation and patient empowerment models have converged into the novel health navigator model. This model was co-designed with input from both people experiencing homelessness and health and social care professionals working with them as well as those working in primary care settings. The project piloted this model in 4 European countries to assess its effectiveness in connecting people experiencing homelessness with cancer prevention services. To the authors’ knowledge, CANCERLESS is the first multicenter study involving people experiencing homelessness from different countries, marking substantial progress in research within this field. However, challenges such as data variability, missing data (missing completely at random and missing not at random), and fragmented in care may introduce biases in the results [20,21].

Building on the premise that various studies have highlighted the inherent heterogeneity within this population [22,23], this diversity becomes even more pronounced when research conducted in different geographical and cultural contexts is considered. While such variability is evident, it should not be regarded solely as an intrinsic characteristic of the data but must be systematically quantified. This necessity arises because a portion of this heterogeneity may stem from biases introduced either by differences among the population subgroups studied or by external factors unrelated to the participants, such as variations in the conditions under which the interventions were conducted. If left unaddressed, this heterogeneity could lead to erroneous interpretations of the results and, consequently, to inaccurate conclusions that undermine the validity of the analysis [24-26]. Several techniques aim to detect variability among samples and approach the data quality (DQ) of datasets [27]. Among them, spatial DQ techniques [28] have been developed to evaluate the differences between the probability distribution function from multiple sources, and these techniques have been demonstrated to deal with sets of heterogeneous data, including multivariate and multitype. Additionally, these are valuable tools for dealing with DQ issues, such as missing values among the datasets, which provide metrics that quantify this heterogeneity and outliers.

Given the above considerations, an exhaustive DQ analysis is needed for several reasons. First, this is key to extracting information about the participants’ characteristic distribution for each pilot site. Second, to examine the differences between these distributions and obtain knowledge about the status of homelessness in different European countries. Finally, these analyses allow us to understand if the information gathered can be used as a different pilot-specific dataset. Thus, this work aims to examine the heterogeneity between the people experiencing homelessness data in 4 different European countries and provide quality metadata while delivering a curated CANCERLESS dataset to extract common knowledge and conclusions.

## Methods

### Dataset Conformation

Data collection was carried out between June 2022 and November 2023. A total sample of 652 people experiencing homelessness was collected: 142 from Austria, 158 from Greece, 197 from Spain, and 155 from the United Kingdom. Participants were included if they were at least 18 years of age and experiencing homelessness, fitting any classification of the European Typology of Homelessness and Housing Exclusion [29]. Participants were excluded if they could not provide informed consent, had difficulties interpreting the aim of the research, did not accept to participate in the study, if they had a known cancer diagnosis or have had cancer previously, and the intervention or study would harm them.

Data used in this study were collected as part of the CANCERLESS project. The study followed a pre- and postdesign, but in this specific analysis, we present only data collected at baseline (T0), that is, before each individual started the intervention. This approach allows us to see the characteristics of each pilot at the beginning of the intervention and, subsequently, to observe the initial differences that will serve to perform a correct analysis of the data.

The questionnaires included in the database were the following: EQ-5D-5L to measure self-reported health-related quality of life [30], Health Care Empowerment Questionnaire (HCEQ) to assess users’ empowerment related to health care [31], Brief Symptom Inventory-18 to measure psychological distress [32], and the Person-Centered Coordinated Care Experience Questionnaire to evaluate several domains of person-centered coordinated care from the perspective of the user [33]. Additionally, specific-purpose questions were included regarding the following information: sociodemographics, health literacy, active diagnosis, medication taken, risk behaviors, healthy lifestyles, and previous use of health care services. Multimedia Appendix 1 provides the complete list of the T0 questionnaires.

The T0 questionnaire was administered to all pilot participants. A coordinator at each pilot site recorded the responses. Later, the information was uploaded by the research team of each pilot to a digital platform created by this research consortium called CANCERLESS Interventions Data Management Application, which stores the data in a digital format. This platform also provides feedback to the coordinator through metrics and aggregated information.

### Methods—Data Quality Workflow

**Figure 1:**
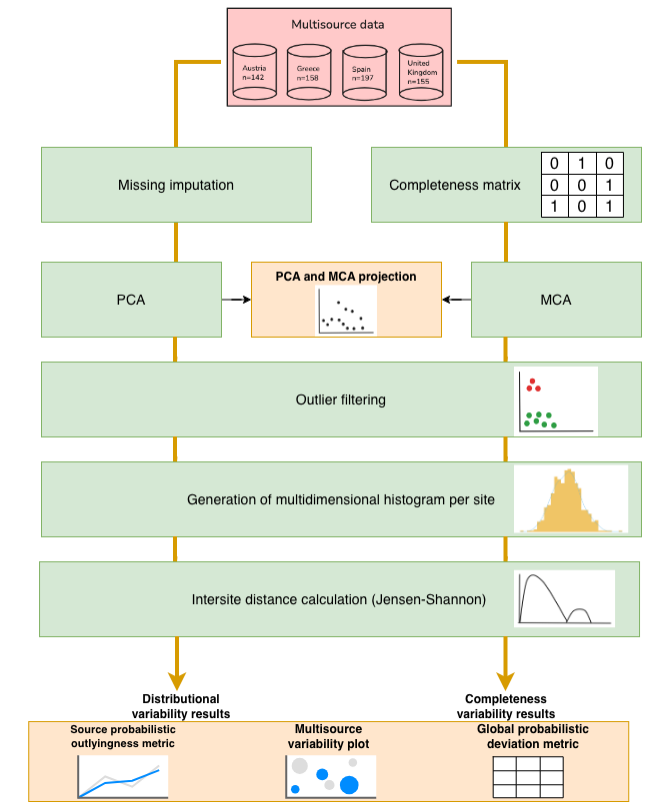
Overview of the methodology to obtain data quality metrics for the CANCERLESS dataset. Two types of analysis are conducted, with shared steps. For the complete dataset, missing values are imputed using iterative imputation techniques. In the missing data analysis, a matrix is built with values indicating 0=ordinary, 1=dependent, or 2=nonmissing. Dimensionality reduction is applied: PCA for complete data and MCA for missing data. After preprocessing, histograms for each country are normalized and combined to calculate distances from the centroid, yielding global probabilistic deviation, source probabilistic outlyingness, and multisource variability values. For specific dataset sections, the same process is applied to each subset. MCA: multiple correspondence analysis; PCA: principal component analysis.

Figure 1 shows the methodology followed in this study. The workflow is divided into 2 blocks for assessing the intersite coherence, one across the information captured in the questionnaires and the other across their completeness patterns. The main difference lies in the preprocessing step: for the questionnaire, imputation is performed, whereas for completeness, a matrix of fault categories is used. Subsequently, a dimensional reduction technique was applied according to the analysis performed—see the specific details in the following subsections. The following steps were common to both analyses: calculating outlier filtering, histogram application to represent the probability density function of the dataset, followed by flattening, normalization, and column stacking, to be used as input for the information theory–based DQ metrics. The technique for evaluating DQ and response variability across the pilot studies involved transforming each dataset into a probability distribution, then applying comparative metrics to measure similarity and identify missing information and therefore the level of nonoverlapping between them. The technique relies on the calculus of a simplex, multidimensional generalization of a triangle, conserving the intersite dissimilarity where the centroid represents an informed average distribution of the sources. Subsequently, 2 metrics were computed, constrained to the Jensen-Shannon distance: the source probabilistic outlyingness (SPO), measuring the dissimilarity of each source to the average distribution; and the global probabilistic deviation (GPD), gauging the degree of global variability among the sources [34,35]. The derived metrics, that is, GPD and SPO, are bounded between 0 and 1, so 0 means equal distributions and 1 means nonoverlapping. For the SPO, a value of 0 denotes the closest possible approach to the centroid, while for the GPD, it indicates the least data variability. Finally, visual plots of these metrics are shown to compare the variability results in a visual form between the different pilots. All these steps were later used to extract GPD, SPO, and multisource variability (MSV) for the global dataset and each section inside the questionnaire.

### Questionnaire Information

This approach has been used following a top-down methodology analyzing multivariate to univariate data, that is, to compare the whole T0 questionnaire and its sections. We excluded textual and multiple-choice options from the analysis since there was a high number of missing data and a much smaller number of category selections per question than the total number of choices, implying minimal variability with no final effect on the analysis. First, we preprocessed the data to transform categorical text into numerical variables along with an iterative imputation method [36,37]. This method performs multivariate imputation through chained equations. Initially, all missing values are filled with simple univariate estimates (eg, the feature’s mean). Thereafter, each incomplete variable is regressed on the other features using a Bayesian ridge estimator to predict its missing entries. Predicted values replace the initial estimates, and this process cycles through all variables for a predefined number of iterations or until convergence of the imputed values is reached.

Subsequently, to obtain a multivariate representation, we applied principal component analysis (PCA) [38] to reduce the dimensionality of the original 130-variable space to 3 dimensions and select the top 20 variables with the highest explained variance. Following this, we segregated the data by pilot source and conducted outlier filtering using the local outlier factor method [39], removing outliers specific to each pilot site. Finally, we computed a 3D histogram for each pilot, using 10 bins for each dimension, which were then used as the input in the method.

### Completeness Patterns

Regarding the comparison of patterns related to blank responses or missing data, the T0 questionnaire comprises questions with varying levels of branching—specific queries are intended to be answered only if certain conditions from prior responses are met (eg, the question about the number of cigarettes smoked daily is only relevant if the participant declares themselves as a smoker in the preceding questions). Consequently, we distinguished between “not applicable missing” and ordinary missing data, which was applied using a predefined rule set. Once the rule was defined, it was applied to the dataset, obtaining a missing matrix and transforming the values into one of these categories. After the transformation, we applied the multiple correspondence analysis (MCA) [40] technique to reduce the dimensionality of the data and select the top 20 variables with the highest explained variance, thus identifying the most influential questionnaires since MCA is a specific technique to reduce dimensionality for categorical values. Next, the local outlier factor methodology was applied to remove outliers over the data on the reduced space. Finally, the multidimensional histogram that was flattened and normalized to be used as GPD’s input was created.

### Exploratory Analyses

The exploratory analyses of the study are presented using the following methods. First, biplots with 2 dimensions are generated after the original data undergo dimensionality reduction either using PCA or MCA. Additionally, the GDP methodology incorporates the MSV plot [35], where the 2 or 3 components with the highest variances are projected using multidimensional scaling. In the MSV plot, sources are represented as circles or spheres, and the distance between them indicates the distance between their distributions, based on the Jensen-Shannon distance, with the circle’s radius representing the number of cases.

To conduct this analysis, we used the Python 3 programming language [41] and the NumPy, pandas, scikit-learn, and prince libraries [42-45]. The experimentation notebooks, including a Python version of the MSV metrics and visualizations, are available on GitHub [46].

### Ethical Considerations

The study was approved by the ethics committee of the Medical University of Vienna (1702/2021), which served as the lead ethics committee for the project. Additionally, each pilot site in the 4 countries secured approval from their respective ethical review boards or institutions before commencing data collection, and Universitat Politècnica de València also obtained approval from an ethics committee, specifically for the management of participants’ personal data. Prior to conducting any data collection, participants were given an information leaflet detailing the study’s purpose and were allowed to ask questions to help them decide on their participation. It was emphasized to all participants that their involvement was entirely voluntary, and they could opt out of answering any questions that made them uncomfortable. Participants were explicitly reassured that declining participation would not negatively impact their access to health and social care services. Informed consent was obtained from all participants through signed forms, which were also verbally confirmed at the beginning of the data collection sessions. All collected data have been securely stored in accordance with data protection regulations. CANCERLESS Interventions Data Management Application platform securely stores data in agreement with the General Data Protection Regulation, following strict guidelines for collecting, processing, storing, and transferring personal data in the European Union and the European Economic Area. Participants did not receive any financial compensation for taking part in the project.

## Results

### Overview

Data used in this study comprehend the whole T0 set of responses, whose demographic distribution is presented in Table 1.

**Table 1:** Demographic distribution among the different pilots.

Pilot	Values, n (%)	Age (years), mean (SD)	Male, n (%)	Female, n (%)	Nonbinary, n (%)	Other, n (%)	Do not want to disclose sex, n (%)
Austria	142 (21.8)	45.28 (12.83)	60 (42.3)	82 (57.7)	0 (0)	0 (0)	0 (0)
Greece	158 (24.2)	51.57 (13.53)	121 (76.6)	34 (21.5)	0 (0)	2 (1.3)	1 (0.6)
Spain	198 (30.3)	48.94 (14.82)	110 (55.6)	87 (43.9)	0 (0)	0 (0)	0 (0)
United Kingdom	155 (23.8)	42.91 (12.04)	127 (81.9)	27 (17.4)	1 (0.6)	0 (0)	0 (0)
Total	652 (100)	47.87 (13.06)	418 (64.1)	230 (35.3)	1 (0.2)	2 (0.3)	1 (0.2)

### Questionnaire Data Analysis

**Figure 2:**
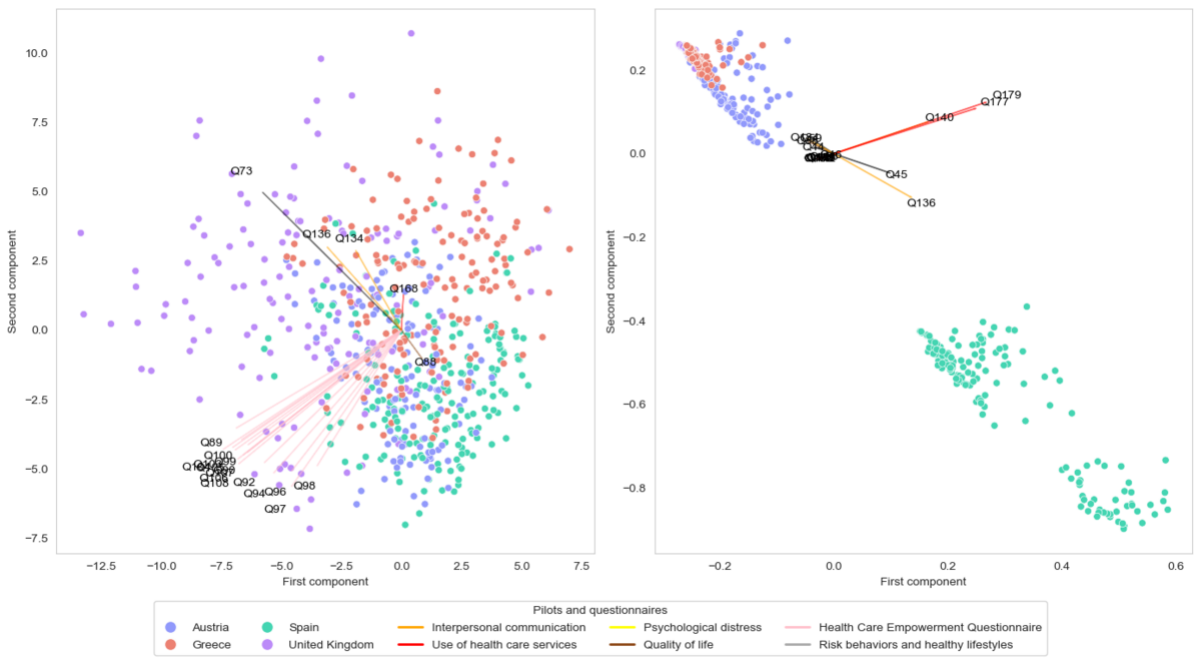
Scatter plot of data along the 2 principal components elicited from the principal component analysis and the multiple correspondence analysis, respectively. Each point is colorized depending on its pilot precedence. Most contributed questions for the principal component analysis calculation have been shown with arrows, depending on the questionnaire they belong to. Tables S1 and S2 in Multimedia Appendix 2 present a detailed table of both, indicating the specific questions and their associated questionnaires. For the global analysis, the principal component analysis indicates a central point where most of the samples are concentrated, with the UK pilot showing the greatest dispersion relative to the others. There is a substantial difference between the UK and Spanish pilot samples. In contrast, Austria and Greece pilots show a wider distribution of their samples. In the completeness case, the figure clearly shows 2 different parts, being the pilots from the United Kingdom, Greece, and Austria quite similar compared to Spain, where it presents an entirely different type of missing from the other pilots, being interpersonal communication and use of health care services sections the most relevant in the calculation of the multiple correspondence analysis. Q: question.

The left side of Figure 2 shows the scatter plot of the 2 first PCA components of the complete dataset for the entire questionnaire. The UK pilot shows a larger variance in comparison to the others. In addition, substantial differences in position and sparsity can be appreciated between the different pilot sites. The PCA loadings in the figure show the most critical variables that have influenced the PCA, which are divided into sections. The HCEQ, which is the one with the greatest dispersion, is also the most influential, as it is the questionnaire with the highest contribution to the PCA with questions Q89, Q90, Q92, Q94, Q96, Q97, Q98, Q99, Q100, Q103, Q104, Q105, Q106, Q107, and Q10, followed by the interpersonal communication questionnaire with Q134 and Q136, afterward use of health care services with Q168, quality of life with Q88, and psychological distress with Q73. Table S1 in Multimedia Appendix 2 provides detailed information regarding the questions corresponding to the selected numbering.

**Figure 3:**
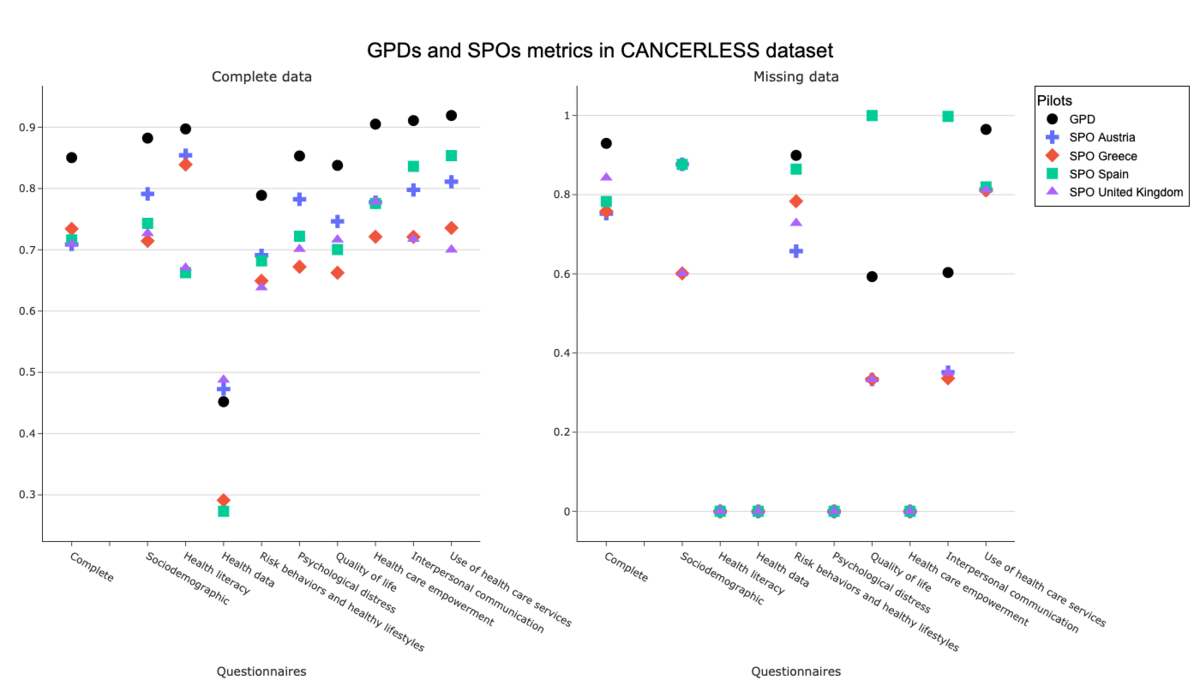
Results from the GDP and SPO multicentric analysis were applied to both the complete dataset (left side) and the completeness (right side) from the dataset composed of the T0 interviews in Austria, Greece, Spain, and the United Kingdom. The SPO indicates the distance of that pilot from the latent central tendency; the GPD can be understood as the mean of the variability of each pilot; a value close to 0 means less variability. In our case, a value of 0 indicates no missings for these questionnaires. Generally, a large variability is observed, especially in the Spanish pilot. GPD: global probabilistic deviation; SPO: source probabilistic outlyingness.

The left side of Figure 3 shows the resultant DQ metrics, which generally present a wide dispersion between pilots for the overall questionnaire and most of the questionnaires. Noteworthy, the health data questionnaire shows metrics less than 0.5 observed, in contrast to those in the other questionnaires with values exceeding 0.8, indicating a clear difference for each pilot. For health care services, interpersonal communication, and health care empowerment, the GPD value exceeds 0.9, highlighting the divergence shown in Figure 2. However, several imputations have been performed for the case of Spain in the interpersonal communication and use of health care services sections.

The overall analysis of the questionnaire reveals 3 types of variability patterns. First, we found questionnaires with very different distributions between pilots. Still, there are no decisive questions within the questionnaire, as they do not appear on the left side of Figure 2 as the most relevant in the PCA, such as sociodemographic data, health literacy, and risk behaviors and healthy lifestyles. Second, questions related to health data showed fewer differences concerning the pilots compared to the other questionnaires, which had a GPD of 0.452. Finally, questionnaires such as psychological distress, quality of life, HCEQ, interpersonal communication (Person-Centered Coordinated Care Experience Questionnaire), and the items related to the use of health care services, apart from diverging between the different pilots according to the metrics applied, present some questions that are determinant and differentiable by country.

### Missing Patterns Analysis

From the 652 participants analyzed and the 130 questions collected at T0, a total of 84,760 values were obtained. Of these, 70,384 of 84,760 (83%) values are complete and valid, 6260 of 84,760 (7.4%) values are not applicable missing, and 8116 of 84,760 (9.6%) values are ordinary missing.

The right side of Figure 2 shows the MCA projection of the missing profiles to 2 dimensions, where a cluster of Austria, Greece, and the United Kingdom can be observed at the top-left side of the plot. Mainly, Spain samples can be observed at the bottom-right of the figure, denoting that Spain is very different from the other sites in terms of the missing pattern. In this instance, the questionnaire with the highest number of questions was the use of health care services, comprising Q155, Q158, Q167, Q161, Q152, Q180, Q176, Q179, and Q178. This was followed by the interpersonal communication questionnaire, which included Q138, Q142, Q140, Q136, and Q134. Next was risk behaviors and healthy lifestyle, encompassing Q45, Q46, Q44, and Q59, and quality of life with Q88. Table S2 in Multimedia Appendix 2 provides detailed information regarding the questions corresponding to the selected numbering.

The right side of Figure 3 offers both a global and section-by-section view of missing-data behavior after applying the GPD and SPO metrics. Overall variability is high; yet, 3 distinct patterns emerge. First, several sections—health literacy, health data, psychological distress, and health care empowerment—show virtually no disparity across pilots because they contain no missing values, so their GPD and SPO scores sit near 0. Second, marked differences appear in sociodemographic data, risk behaviors, healthy lifestyles, and use of health care services, where some pilots contribute many missings, while others contribute few.  Third, a distinct Spain-specific pattern emerges. Spain is the only pilot exhibiting substantial variability that elevates the mean GPD in sections where the remaining pilots show minimal variability—particularly in quality of life (Spain: 100/1576, 6.3% missing; others combined: 10/3640, 0.3% missing) and in interpersonal communication, where the SPO profile departs markedly from the consistently low missingness observed elsewhere.

## Discussion

### Summary of Main Findings

This study investigated heterogeneity among people experiencing homelessness in 4 European countries and evaluated the quality of data collected in a multicenter cohort. Understanding variability in demographics, health status, and service use is essential for designing interventions tailored to this population’s needs. The analysis showed pronounced between-pilot differences: for most questionnaires, the GPD exceeded 0.7 at both pilot and aggregate levels (Figures S1 and S2 in Multimedia Appendix 3), indicating substantial heterogeneity. The sole exception was the health questionnaire, whose lower GPD likely reflects that only a single quantitative item remained after discarding multichoice and free-text responses. Missing-data diagnostics revealed further site-specific discrepancies, with the Spanish cohort diverging most markedly, particularly in the use of health services, quality of life, and interpersonal communication (Figures 2 and 3).

Participants’ responses reveal notable differences in the experiences of people experiencing homelessness across the various countries. While the heterogeneity observed within the homeless population can, in part, be attributed to country-specific factors, such as distinct socioeconomic and health care contexts, variations in responses may also stem from differences in the implementation of interventions and the subjective perceptions of individual participants. These discrepancies are particularly evident in key areas assessed by the PCA, including health-related domains, where divergent patterns emerge based on both contextual and personal factors. In the case of Q136: “Do you have a care plan (or a single plan of care) that takes into account all your health and wellbeing needs?” Spain stands out as the only country, where more individuals have health plans than those without. Additionally, Austria is unique in having no instances of “I don’t know” responses to this question. For Q134: “Do you have a single professional (or several professionals) who takes responsibility for coordinating your care across the services that you use?” Spain and the United Kingdom are distinguished by having more positive responses than negative ones. Notably, the United Kingdom records the highest number of participants uncertain about their answers. Furthermore, Spain shows a substantially higher number of blank responses for both Q136 and Q134. Meanwhile, concerning 3 questions belonging to the HCEQ subquestionnaire: Q104: “During the last 6 months, how important is it that you obtained all the information you wanted?” Q106: “During the last 6 months, how important is it that you and your loved ones decide the need for the health care and services?” and Q108: “During the last 6 months, how important is it that you and your loved ones decide the amount of health care and services?” In general terms, both in the United Kingdom and Austria, the response rate is higher than in Spain and Greece. Meanwhile, the United Kingdom and Austria maintain the same number of answers for these questions, with the UK participants being the most important for all 3 questions and the Austrian participants the second most important. We see how, in the case of Greek cities and Spain, the difference lies in the type of question; in the case of Q106, the Greek cities considered it very unimportant, while the participants in the Spanish pilot considered it the most important of the 3. However, the opposite effect was observed in the case of Q104 and Q108. Finally, for Q88: “We would like to know how good or bad your health is today. 100 means the best health you can imagine. 0 means the worst health you can imagine.” being the perceived health status is a major patient-reported experience measure of the study.

### Significance

This study, as far as the authors know, is the first study confirming the heterogeneity within the group of people experiencing homelessness in a European multicenter study, as most of the previous studies have been primarily focused on the US-American context [22,23,47]. The study’s results enable quantifying this heterogeneity between the different pilots that conform to the database through various factors that should be considered in a posteriori analysis. First, despite heterogeneity, the only set of items that can be assumed to be the most appropriate if a global analysis is desired is the health data questions since they do not present any missings, as are the ones with the least variability between pilots. Second, in the case of interpersonal communication and use of health care services, the global result could not be correctly determined since most of the data were imputed in the case of Spain, which does not make the analysis at a global level appropriate. Finally, the analyses at the section level should be done separately for each pilot, with the HCEQ being the most relevant since it presents greater variability between pilots and does not contain missing values. Therefore, the analysis of the pilots must be carried out independently, and the conclusions of one pilot cannot be extrapolated to the others, given the great divergence found between them. The disparities in the global dataset are more pronounced for Spain. In contrast to the other pilots, the methodology shows that ordinary missing values constitute the majority, especially within the interpersonal communication and health care utilization questionnaires.

In this context, it is important to recognize that eliciting such variation, whether through the proposed methodology or other approaches, is not necessarily a DQ issue. Rather, it is an expected step for adapting the interventions to the specific socioeconomic and health care contexts. However, the nature and source of this variation should be carefully considered, as concerns may arise if the variation is driven by systematic measurement error or bias, which could influence the validity of the findings and the effectiveness of the interventions. This proposed methodology can help to find such variation prior to any analysis and therefore approach it, considering the characteristics of the dataset under study.

### Strengths and Limitations

Including this methodology to analyze the data enables us to demonstrate 3 objectives that help ensure DQ for improved analysis. First, our approach allows us to evaluate the variability existing in the data, especially the differences between the data from different sources from a more visual perspective and with a single iteration, considering all types of variables for the analysis, without going into detail in classical tests that are more sensitive to the analysis. The technique used for DQ of the responses in the different pilots and to evaluate their quality in terms of missing information makes use of the comparative datasets, transforming them into probability distributions that allow using comparative metrics of similarity and, therefore, the level of nonoverlapping between them. Thus, it is necessary to know the boundaries regarding the exploitation capacity of the dataset for prospective uses, especially with the scarcity of homelessness quantitative information. Second, this hierarchical approach allows for monitoring the pilots’ progress and comparing them as a whole and by different sections. As data become available, we can promptly detect and correct deviations from the established methodology or protocol. This approach also enables the systematic identification of patterns and causes of missing data, supports the implementation of targeted corrective measures, and—by embedding these methods in a control framework—drives continuous improvement in information quality. Third, further analyses of DQ with new data or batches can be applied thanks to the replicability of the methodology, given that the source code has been made public.

The analysis of the overall results is affected by the large number of missing values in the Spain dataset. Since there are many questions with missing data, an imputation had to be performed, which negatively influenced the results. In addition, the number of samples is not very high for our applied method, which may also impact the results. This is because the methodology used is sensitive and requires a large amount of data to generalize results more accurately. Missing values were imputed with reference to the pooled empirical distribution of all pilot sites. While this cross-site approach may introduce some location-specific bias, it enhances methodological robustness by preserving the overall variance structure; this was exemplified in the Spanish cohort, where the 2-part analysis coupled with the imputation procedure successfully pinpointed the sources of variability.

### Future Perspectives

For all of those reasons, the results of this study will facilitate prospective dataset DQ labeling for prospective publication in related health data access bodies in the framework of the European Union’s European Health Data Space (EHDS) initiative [48] in compliance with Article 78 on DQ that will allow secondary use of health data for research, innovation, and policymaking under conditions of security and privacy by ensuring a certain level of DQ that will enable users to know in advance the characteristics of the CANCERLESS dataset thanks to the applied methodology.

Following current best-practice guidance on dataset curation, other DQ dimensions not covered in this study—such as temporal coherence, consistency, or correctness—will be assessed as part of the mid-scale pilot of the EHDS project QUANTUM (“Quality, Utility and Maturity Measured”). QUANTUM will pilot an European Union–wide framework for rating both the intrinsic quality of health datasets and the maturity of their curation processes; CANCERLESS database will conform to one of these analyses, supporting our analysis and getting externally validated, a multidimensional appraisal that complements this analysis.

### Conclusions

The analysis of this multicenter sample of people experiencing homelessness from 4 different European countries revealed substantial heterogeneity between sites of special importance for health care empowerment, quality of life, and interpersonal communication values, which showed the most differences between pilots. Particularly, differences in missingness patterns in data were larger than the ones found on the raw data, especially for the Spain pilot, highlighting that the interpersonal communication, health care empowerment, and use of health services data present a high number of nulls and should not be used for analysis. Further qualitative techniques are needed to understand the cause of this deviation from the other pilots. This methodology should be applied to multicentric data to monitor a pilot as the data become available and, therefore, amend possible protocol deviations. In summary, as quantitative data about homelessness are scarce, using a DQ-validated dataset in the context of the EHDS is crucial to ensure the reliable use of data to facilitate research on vulnerable populations such as people experiencing homelessness.

## Appendix

Multimedia Appendix 1 T0 questionnaire.

Multimedia Appendix 2 Most principal component analysis and multiple correspondence analysis relevant questions.

Multimedia Appendix 3 Multiple correspondence analysis metrics for CANCERLESS dataset.

## Abbreviations

DQ: data quality
EHDS: European Health Data Space
GPD: global probabilistic deviation
HCEQ: Health Care Empowerment Questionnaire
MCA: multiple correspondence analysis
MSV: multisource variability
PCA: principal component analysis
SPO: source probabilistic outlyingness
