# Supplementary material for: Multisource Coherence Analysis of the First European Multicenter Cohort Study for Cancer Prevention in People Experiencing Homelessness: Data Quality Study
Source: JMIR Med Inform. 2025 Nov 14;13:e73596. doi: 10.2196/73596 (PMC12663700; doi:10.2196/73596)
Supplement: Multimedia Appendix 2 [file medinform_v13i1e73596_app2.docx]

Table S1 lists the 20 most relevant questions in the PCA calculation for the complete dataset, while Table S2 shows the 20 most relevant questions for the MCA calculation. In both tables, the first column indicates the numbering used in the article, the second column provides the question text, and the third column identifies the corresponding questionnaire.

**Table S1**. Most 20 relevant questions in PCA for complete CANCERLESS dataset.

| Questionnaire |  | Question number |
| --- | --- | --- |
|  |  |  |
| Health Care Empowerment |  |  |
|  | During the last 6 months did you feel that you asked for explanations? | Q89 |
|  | During the last 6 months did you feel that you asked questions? | Q90 |
|  | During the last 6 months did you feel that you were able to talk to a professional? | Q92 |
|  | During the last 6 months did you feel that you obtained all the information you wanted? | Q94 |
|  | During the last 6 months did you feel that you and your loved ones decide the need for the health care and services? | Q96 |
|  | During the last 6 months did you feel that you and your loved ones decide the type of health care and services? | Q97 |
|  | During the last 6 months did you feel that you and your loved ones decide the amount of health care and services? | Q98 |
|  | During the last 6 months how important is it that you asked for explanations? | Q99 |
|  | During the last 6 months how important is it that you asked questions? | Q100 |
|  | During the last 6 months how important is it that your choices were respected? | Q103 |
|  | During the last 6 months how important is it that you obtained all the information you wanted? | Q104 |
|  | During the last 6 months how important is it that you got the help you needed? | Q105 |
|  | During the last 6 months how important is it that you and your loved ones decide the need for the health care and services? | Q106 |
|  | During the last 6 months how important is it you and your loved ones decide the type of health care and services? | Q107 |
|  | During the last 6 months how important is it that you and your loved ones decide the amount of health care and services? | Q108 |
| Use of Health Care Services |  |  |
|  | How many times have you been vaccinated from the Papilloma virus? | Q168 |
| Psychological Distress |  |  |
|  | Feeling worthless | Q173 |
| Quality of Life |  |  |
|  | We would like to know how good or bad your health is today. 100 means the best health you can imagine. 0 means the worst health you can imagine. | Q88 |
| Interpersonal Communication |  |  |
|  | Do you have a single professional (or several professionals) who takes responsibility for coordinating your care across the services that you use? | Q134 |
|  | Do you have a care plan (or a single plan of care) that takes into account all your health and wellbeing needs? | Q136 |

**Table S2**. Most 20 relevant questions in MCA for completeness part in CANCERLESS dataset.

| Questionnaire |  | Question Number |
| --- | --- | --- |
| Use of Healthcare Services |  |  |
|  | To the extent of your knowledge have you been vaccinated from the Hepatitis A? | Q155 |
|  | To the extent of your knowledge have you been vaccinated from the Hepatitis B? | Q158 |
|  | To the extent of your knowledge have you been vaccinated from the Papilloma virus? | Q167 |
|  | To the extent of your knowledge have you been vaccinated from the Covid-19? | Q161 |
|  | In the past 12 months, how many times did you visit an oncologist (at a hospital’s outpatient department)? (Do not include visits while in a hospital or to a hospital’s Accident and Emergency Department) | Q152 |
|  | How many total nights did you spend in a hospital in the past 12 months? | Q180 |
|  | In the past 12 months, how many times did you visit a social/support worker? (Do not include visits while in a hospital or to a hospital’s Accident and Emergency Department) | Q176 |
|  | In the past 12 months, how many times were you urgently admitted into a hospital? | Q179 |
|  | How many different times did you stay in a hospital overnight or longer in the past 12 months? | Q178 |
| Interpersonal Communication |  |  |
|  | Is this care plan (or plan of care) available to you? | Q138 |
|  | To what extent do all the professionals involved in your care appear to be following the same care plan (or plan of care)? | Q142 |
|  | To what extent have you found your care plan (or plan of care) USEFUL FOR YOU to manage your health and wellbeing? | Q140 |
|  | Do you have a care plan (or a single plan of care) that takes into account all your health and wellbeing needs? | Q136 |
| Risk Behaviours and Healthy Lifestyles |  |  |
|  | In the past 30 days, have you had sexual intercourse with someone? | Q44 |
|  | How many of those partners did you have sex with at least once without a condom? | Q46 |
|  | How many different partners have you had sex with within the last 30 days? | Q45 |
|  | Do you usually share clothes, towels or bedding with other people? | Q46 |
| Quality of Life |  |  |
|  | We would like to know how good or bad your health is today. 100 means the best health you can imagine. 0 means the worst health you can imagine. | Q88 |
