# Supplementary material for: Multisource Coherence Analysis of the First European Multicenter Cohort Study for Cancer Prevention in People Experiencing Homelessness: Data Quality Study
Source: JMIR Med Inform. 2025 Nov 14;13:e73596. doi: 10.2196/73596 (PMC12663700; doi:10.2196/73596)
Supplement: Multimedia Appendix 3 [file medinform_v13i1e73596_app3.docx]

The data presented in Figures S1 and S2 applies the MSV method to the CANCERLESS dataset, both to the complete dataset and to the completeness of the data.


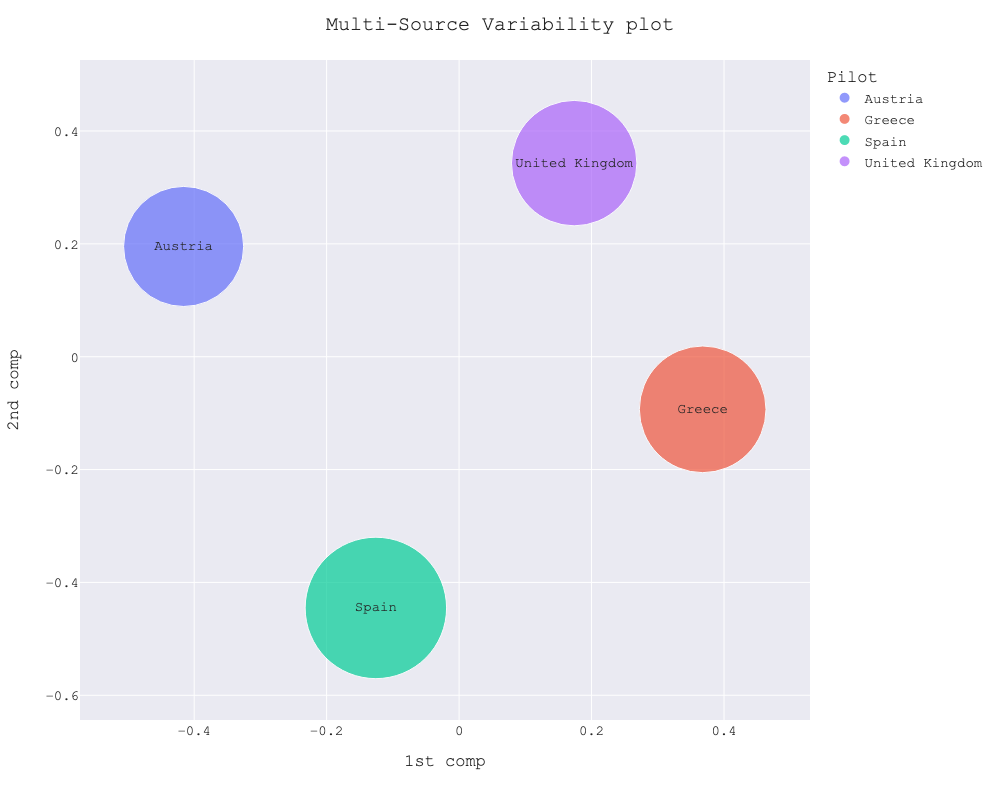


**Figure S1.** Multi-Source Variability (MSV) plot from the data analysis of the CANCERLESS project following the algorithm described in Figure 1. Differences between Austria, Greece and United Kingdom are notable, there is a clear divergence between the different pilots.

Figure S2 illustrates the MSV plot resulting from the missing part. The figure reinforces the discrepancy observed in the MCA. It is highlighted that the missing data from the UK, Austria and Greece pilots have similar characteristics, while those from Spain differ significantly from the previous ones. Since the algorithm calculates the distance based on the global mean, the distance of Spain with respect to the other pilots is not larger since the divergence of Spain significantly influences the determination of the center of the intervention. However, it is evident that the representation of the Spanish data follows a completely different direction than the others due to its characteristics.


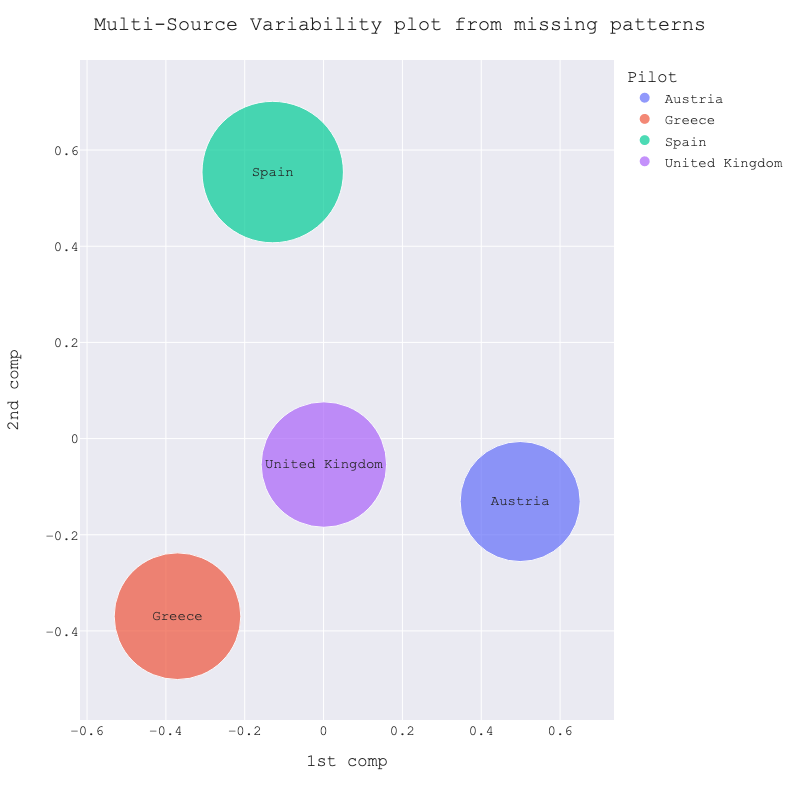


**Figure S2.** MSV plot for the analysis of missing patterns in two dimensions. The position of the centers is determined by GPD algorithm while the size of each circle is proportional to the number of participants in each pilot. Spain's difference from the other pilots shows a variety in the types of missings for that pilot.
